# Cross-Sectional Associations of Active Transport, Employment Status and Objectively Measured Physical Activity: Analyses from the National Health and Nutrition Examination Survey

**DOI:** 10.1136/jech-2017-210265

**Published:** 2018-05-05

**Authors:** Lin Yang, Liang Hu, J. Aaron Hipp, Kellie R. Imm, Rudolph Schutte, Brendon Stubbs, Graham A. Colditz, Lee Smith

**Affiliations:** 1 Department of Epidemiology, Center for Public Health, Medical University of Vienna, Austria; 2 Division of Public Health Sciences, Washington University School of Medicine, MO, USA; 3 Department of Sport Science, Zhejiang University, Hangzhou, China; 4 Center for Geospatial Analytics, Department of Parks, Recreation and Tourism Management, North Carolina State University, USA; 5 Department of Preventive Medicine, Keck School of Medicine, University of Southern California, USA; 6 Department of Medical Science and Public Health, Faculty of Medical Science, Anglia Ruskin University, Chelmsford, UK; 7 Physiotherapy Department, South London and Maudsley NHS Foundation Trust, Denmark Hill, London, SE5 8AZ, UK; 8 Health Service and Population Research Department, Institute of Psychiatry, Psychology and Neuroscience, King's College London, De Crespigny Park, London, Box SE5 8AF, UK; 9 Faculty of Health, Social Care and Education, Anglia Ruskin University, Chelmsford, UK; 10 Faculty of Science & Technology, Sports & Exercise Sciences, Anglia Ruskin University, Cambridge, UK

**Keywords:** Active transport, moderate to vigorous intensity physical activity, employment status, accelerometer

## Abstract

**Background:**

To investigate associations between active transport, employment status and objectively measured moderate-to-vigorous physical activity (MVPA) in a representative sample of US adults.

**Methods:**

Cross-sectional analyses of data from the National Health and Nutrition Examination Survey. A total of 5180 adults (50.2 years old, 49.0% men) were classified by levels of active transportation and employment status. Outcome measure was weekly time spent in MVPA as recorded by the Actigraph accelerometer. Associations between active transport, employment status and objectively measured MVPA were examined using multivariable linear regression models adjusted for age, BMI, race and ethnicity, education level, marital status, smoking status, working hour duration (among the employed only), and self-reported leisure time physical activity.

**Results:**

Patterns of active transport were similar between the employed (n=2,897) and unemployed (n=2,283), such that 76.0% employed and 77.5% unemployed engaged in no active transport. For employed adults, those engaging in high levels of active transport (≥90 min/week) had higher amount of MVPA than those who did not engage in active transport. This translated to 40.8 (95% CI: 15.7, 65.9) additional minutes MVPA per week in men and 57.9 (95% CI: 32.1, 83.7) additional minutes MVPA per week in women. Among the unemployed adults, higher levels of active transport were associated with more MVPA among men (44.8 min/week MVPA, 95% CI: 9.2, 80.5), only.

**Conclusions:**

Findings from the present study support interventions to promote active transport to increase population level physical activity. Additional strategies are likely required to promote physical activity among unemployed women.

## Introduction

Regular and sustained participation in physical activity is associated with better physical and mental health and is associated with healthy ageing in adults.^1-3^ Consequently, the global physical activity recommendations developed by the World Health Organization recommends that adults should do at least 150 minutes (2 hours and 30 minutes) a week of moderate-intensity, or 75 minutes (1 hour and 15 minutes) a week of vigorous-intensity aerobic activity, or an equivalent combination of moderate- and vigorous-intensity aerobic activity.^4^ However, despite these recommendations, population levels of physical activity in the US are low with just 51.7% of adults meeting the guidelines in 2016.^5^ To date, efforts to increase population levels of leisure or occupational physical activity have yielded limited success particularly over the long term.^6, 7^

Active travel or active transport (walking and bicycling for transportation) may provide an alternative opportunity for physical activity. A recent British study^8^ in a sample of 1628 adults showed that changes in active transport were associated with commensurate changes in total physical activity. Compared with those whose active transport remained unchanged, total physical activity decreased by 176.9 min/week in those whose active transport had decreased (adjusted regression coefficient −154.9, 95% CI: −195.3 to −114.5) and was 112.2 min/week greater among those whose active transport had increased (adjusted regression coefficient 135.1, 95% CI: 94.3 to 175.9). Active transport is associated with a range of health outcomes^9^ and has recently been shown to associated with lower risk (0.73 walking, 0.54 cycling) of CVD incidence, the leading cause of death worldwide,^10^ and lower risk of CVD mortality (0.48 cycling, 0.64 walking).^11^ To the best our knowledge, similar data for the US population does not exist. It is possible that by transforming routine daily living into an opportunity for physical activity, active transport overcomes many of the traditional barriers to engaging in leisure-time or occupational physical activity.

A previous systematic review^12^ investigating the association between active travel and physical activity reported mixed results across 15 studies; five studies found associations in the expected direction (more active transport associated with more physical activity), nine found such associations in at least one gender group, and one reported no associations. One potential reason for conflicting results and an important factor that may influence the relationship between active travel and physical activity is employment status. Indeed, available evidence shows that adults from a lower socio-economic-status (a proxy for unemployment) have lower levels of overall physical activity.^13, 14^ In addition, physical activity patterns vary among different geographic regions, such that unemployment itself was associated with lower levels of physical activity in the US population^15^ but not in Sweden^16^ or Canada.^17^ However, it should be note that the Canadian study was carried out over 20 years ago. To the authors' knowledge more recent data does not exist.

Interestingly, recent studies carried out in Canadian population based samples have investigated neighborhood walkability in relation to physical activity. One population based study reported significant associations of higher walkability with higher overall physical activity in the adult population after adjustment for important confounding variables, including employment status.^18^ This association is likely driven by higher transport walking.^19^

Nevertheless, to the best of our knowledge, no study has investigated the relationship between active transport and physical activity stratified by employment status. The employed individual may have greater opportunity for active transport (to and from work), as well as active transport to other destinations (e.g. shop on lunch break). The unemployed individual is likely to have less structure in his/her life^20^ and will not have the opportunity to use active transport to and from work. This potential lower level of active transport may yield lower levels of total physical activity and increase the risk of non-communicable disease risk factors and poorer mental health.^21, 22^ The aim of the present analysis is to investigate the association between active transport and accelerometer measured physical activity in a national representative population sample from the US National Health and Nutrition Examination Survey (NHANES). We hypothesize that those who are employed and use active transport will engage in higher levels of physical activity.

## Methods

### Study Population

The NHANES was designed to provide cross-sectional estimates on the prevalence of health, nutrition, and potential risk factors among the civilian non-institutionalized U.S. population up to 85 years of age.^23^ In brief, NHANES surveys a nationally representative complex, stratified, multistage, probability clustered sample of about 5,000 participants each year in 15 counties across the country. Survey participants were asked to attend a physical examination in a mobile examination center (MEC) or in the participants' home. The present analysis aggregated data from waves 2003-2004 and 2005-2006. During these waves objective physical activity assessment was implemented in the NHANES participants by fitting them with a hip-worn accelerometer (ActiGraph AM-7164) for 7 days. The NHANES obtained ethical approval from the National Center for Health Statistics Research Ethics Review Board and participants provided written informed consent.

We extracted demographic information, employment status and working hour duration, measures of adiposity, smoking history, self-reported leisure time physical activity, self-reported walking and cycling for travel, and objective physical activity and combined them into a single dataset for each data collection wave. We created a single dataset for each wave of data from NHANES 2003-2004 and 2005-2006, and excluded those who were younger than 25 years old, were pregnant, or unable to walk or cycle.

### Accelerometer measured physical activity

NHANES participants were asked during their physical examinations at the MEC to wear an accelerometer (ActiGraph AM-7164, 1 minute epochs) at the right hip for 7 consecutive days to objectively measure free-living physical activity. The ActiGraph AM-7164 is a validated, small lightweight device that provides detailed information about the intensity, frequency and duration of physical activity.^24^ The epoch length was set at 1 minute, and the Actigraph recorded count data for physical activity in the form of counts per minute (cpm). Non-wear time was defined as 60 minutes of consecutive zero counts. A recording of at least 10 hours of data was defined as a valid day, and four or more valid days were required to be included in the analysis. The total minutes of valid data were recorded as the accelerometer wear time. Data on raw moderate-to-vigorous physical activity were calculated from the minutes spent in MVPA bouts (>2020 cpm) of at least 10 minutes. Wear time adjusted MVPA were computed by dividing raw MVPA minutes by total wear time and multiplying the resulting fraction by the average wear time of all participants. We summarized the adjusted total weekly minutes of MVPA for each participant.

### Active transport

Participants self-reported their active transport behavior in the 2003-2004 and 2005-2006 waves. Participants were asked if they “have walked or bicycled as part of getting to and from work, or school, or to do errands?” over the past 30 days. Participants who answered “no” to this question were classified as non-active transporters (zero minutes/week active transport). For those who answered yes, they were further asked about activity frequency (“how often did you do this”), and duration (“On those days when you walked or bicycled, about how long did you spend all together doing this”). Participants who reported spending less than 10 minutes “on these days when you walked or bicycled” were also classified as non-active transporters (zero minutes/week active transport).^25^ Levels of active transport were calculated as the weekly minutes that participants reported participating in walking or cycling. The 30 days' active transport was calculated by multiplying the number of days participants walked or bicycled by their daily duration. We summarized weekly active transport by dividing 30 days' active transport by 30 then multiply by 7 days. Travel mode was defined as non-active transport (zero minutes/week active transport),^25^ low level of active transport (< 90 minutes/week), and high level of active transport (≥90 minutes/week). Ninety minutes was used to approximate the median of the weekly active transport minutes in our study population, which further indicates the suggested minimum amount of weekly physical activity to achieve survival benefit.^26,27^

### Socio-demographic characteristics

Socio-demographic characteristics including age, gender, race and ethnicity, education, marital status, working hour duration, and smoking status were extracted. Based on self-reported race and ethnicity, participants were classified into one of the three racial/ethnic groups: Non-Hispanic White, Non-Hispanic Black, and Hispanic and others. Education levels were classified into four groups: less than 12^th^ grade, high school, some college, and college graduate or above. Participants' marital status were summarized into two groups: live with someone (married, and living with partner), and live alone (widowed, divorced, separated, never married). Based on self-reported occupation, we created a binary variable for employed (working at a job or business, with a job or business but not at work) and unemployed (looking for work, not working at a job or business). For those employed, we further extracted data on their working hour durations. Finally, we classified participants into three groups: never smokers (did not smoke 100 cigarettes and do not smoke now), former smokers (smoked 100 cigarettes in life and do not smoke now), and current smokers (smoked 100 cigarettes in life and smoke now).

### Body mass index (BMI)

Weight and height were measured at the time of physical examinations at the MEC. The measurements followed standard procedures and were carried out by trained technicians using standardized equipment. BMI was calculated as weight in kg/(height in meters)^2^. We categorized study participants into standard BMI categories: underweight (<18.5kg/m^2^), normal weight (18.5-24.9 kg/m^2^), overweight (25.0 – 29.9 kg/m^2^), and obese (≥30.0 kg/m^2^). For analytic purposes, we combined underweight and normal weight participants (≤25 kg/m^2^).^28^

### Self-reported leisure-time physical activity (LTPA)

Using a list of 48 activities, participants self-reported whether they participated in any of these LTPA in the past 30 days, along with the frequencies and durations of these activities. Each activity was coded into a metabolic equivalent task (MET) score based on the 2011 Compendium of Physical Activities, a valid and globally used instrument to quantify the energy expenditure of physical activity in adults.^29^ For each reported activity, MET-minutes per week (MET-min/week) were calculated by multiplying the MET value of each reported activity by the minutes spent in the activity per seven days. Overall LTPA was summarized as the total MET-minutes per week of all reported activities.^26^ Participants were classified as inactive (zero MET-min/week), insufficiently active (<750 MET-min/week), and sufficiently active (≥750 MET-min/week) based on the standard definition.^26^ We used 750 MET-min/week as the threshold because it approximates the amount of MET score for 150 min/week moderate-to-vigorous intensity (3.0-6.0 METs) physical activity that is recommended to adult populations by the World Health Organization.^4^

## Statistical Analysis

Survey analysis procedures were used to account for the sample weights (MEC exam weight), stratification, and clustering of the complex sampling design to ensure nationally representative estimates. We included participants with completed information on accelerometer measured physical activity, active transport behavior, employment status, socio-demographic characteristics, weight, height, smoking, and self-reported LTPA. We calculated the descriptive statistics for participants' characteristics, LTPA categories and accelerometer measured MVPA by their employment status and gender. We summarized weighted means and standard errors for continuous variables, and weighted proportions for categorical variables.

Linear regressions were carried out to quantify associations between levels of self-reported active transport and accelerometer measured MVPA, stratified by employment status. Because of the documented difference in walking and cycling behavior between men and women,^30^ we tested for the interactions of active travel and gender. We then further stratified our analyses by gender provided the significant interaction. The multivariable linear regression models for accelerometer measured MVPA were adjusted for age, BMI, race and ethnicity, education level, marital status, smoking status, self-reported LTPA, and working hour duration among the employed only. We examined the normality of residuals by kernel density estimate and standardized normal probability plots for all the linear regression models. All statistical significance was set at *p*<0.05. All statistical analyses were performed using Stata version 14.0 (STATA Corp., College Station, Texas, USA).

## Results

A total of 5,506 adults aged 25 years or older had sufficient data on accelerometer measured MVPA. Of these, we excluded 218 (5%) participants who did not provide data on active transport or employment status. We further excluded 44 (1%) participants who did not provide information on socio-demographic characteristics or self-reported LTPA. Our study population consisted of 5,180 adults with completed data. The majority of the study population were employed (66.8%). Employed participants' mean age at the time of baseline examination was 44.7 years with mean BMI of 28.5 kg/m^2^, whereas the unemployed participants were older (61.2 years, p<.001) with similar BMI (28.5 kg/m^2^, p=0.96). Accelerometer measured MVPA was significantly higher among those who were employed than the unemployed (51.8 min/week vs. 33.5 min/week, p<.001). Yet, patterns of active transport were similar between the employed and unemployed, such that 76% employed and 77.5% (p=0.3) unemployed engaged in no active transport, and 11.8% employed and 13.2% unemployed achieved 90 or more weekly minutes walking and cycling for travel purpose. We observed statistically significant differences between men and women for most characteristics, except for age among the employed, and education and leisure time physical activity among the unemployed (Table 1).

### Associations between active transport and accelerometer measured MVPA

Table 2 summarizes both the non-adjusted and adjusted associations between levels of active transport and accelerometer measured MVPA in linear regression models. For employed adults, engaging in high levels of active transport (≥90 minutes/week) had higher accelerometer measured MVPA than those who did not engage in active transport in univariate analyses, and these findings were maintained in multivariable analyses. This translated to 40.8 (95% CI: 15.7, 65.9) additional minutes MVPA per week in men and 57.9 (95% CI: 32.1, 83.7) additional minutes MVPA per week in women in the multivariable-adjusted models. Among the unemployed adults, higher levels of active transport were associated with more accelerometer measured MVPA in both men and women in the un-adjusted models. However, in multivariable-adjusted models, the association was only retained among men who engaged in 90 minutes or more active transport per week (44.8 min/week MVPA, 95% CI: 9.2, 80.5).

## Discussion

To the best of our knowledge, the current study is the first to investigate the relationship between active transport and MVPA in a US national representative sample. In this large sample we found that those who were employed and those who were unemployed displayed similar pattern of active and non-active transport. Interestingly, employed men and women who reported using active transport achieved significantly higher levels of MVPA than those who did not use an active transport mode. However, in the unemployed such an association only existed in men.

Findings from the present study support previous literature that has shown associations between the use of active transport and higher levels of physical activity.^8, 12^ However, the systematic review by Wanner et al. (2012)^12^ produced mixed results, potentially due to the lack of considering employment status. For instance, most previous studies investigating the association of active transport and physical activity have either included student population,^31, 32^ or exclusively commuting population.^33-35^ Even among studies that have included adults of mixed employment status, ^36-42^ only one study adjusted for working status in their multivariable logistic regression, which reported a positive association of walking and cycling with physical activity.^42^ Our findings suggest that employment status, particularly among women, is an important factor in the association between active transport and objectively measured MVPA. Future research should consider employment status and an equal distribution of men and women in all groups.

Active transport in adults can yield higher levels of physical activity via active transport per se, an increase in self-efficacy encouraging physical activity in other areas of one's life,^43^ and potentially spontaneous activity en-route. It is likely that employed adults achieved higher levels of physical activity compared to the unemployed, despite similar travel mode distributions, owing to a higher frequency of trips and a more stable pattern of active transport. Employed men and women who used active transport achieved an additional 40.8 and 57.9 minutes of MVPA a week, respectively. This amount of additional MVPA is associated with up to 20% reduction in all cause-mortality and mortality due to CVD and 13% reduction in cancer morality.^44^ Furthermore, this amount equates to 20% and 28% of the total required MVPA for men and women, respectively, which could improve their mental states.^1-3^ These findings encourage the promotion of active transport among the employed who commute via motorized transport.

In the present study unemployed men who use active transport yield higher levels of physical activity than those who use non-active modes. However, this association did not exist in women. According to the US time use survey,^45^ a higher proportion of unemployed men are spending time educating themselves than unemployed women. It is likely that if one is in full-time education they may have a similar commuting routine as to one who is employed and thus will likely have a high frequency of trips and a stable pattern of active transport. Moreover, unemployed women spend a much greater proportion of their day in taking care of the house or family^15^ and thus limiting their discretionary time to travel actively to various destinations. Further research is needed to identify appropriate strategies to promote physical activity among unemployed women.

Key strengths of this study are the large sample representative of the U.S. adult population, objective measures of physical activity and assessment of active transport for any purpose rather than just for commuting. However, the study is not without limitations. The cross-sectional design prohibits attributing causality to the associations between active transport and MVPA. That is, it is not known if active transport results in higher levels of MVPA or if those who have high levels of MVPA are more likely to use active modes of travel. Further investigation using a prospective study design is needed to refute/confirm our results. However, prospective studies in British samples^8^ have shown that a change from non-active to active transport results in higher levels of physical activity. A further limitation involves the use of accelerometers, which are calibrated to record ambulatory activity (hip movement) and may therefore underestimate physical activity undertaken during cycling.

## Conclusion

In this large representative sample of US adults, the distribution of active transport was similar between those who were employed and those who were not. Active transport was associated with higher amount of MVPA in employed men and women and unemployed men. These findings support future interventions to promote active transport to increase population-level physical activity. Additional strategies are likely required to promote physical activity among unemployed women.

## Figures and Tables

**Table 1:** Socio-demographic Characteristics and Physical Activity Levels of Employed and Unemployed Adults Aged 25 Years or Older from the NHANES (2003 - 2006), by Employment Status and Gender

	Employed				Unemployed		
		
		Men	Women	P-value	Men	Women	P-value
2003-2006	N	1,662	1,235		1,017	1,266	
	Weighted N^a^	43,458,242	36,306,134		15,204,399	24,454,478	
Age (year)	Mean (s.e.)	44.4 (0.4)	45.0 (0.4)	0.26	62.1 (0.8)	60.6 (0.7)	0.04
BMI				<.001			<.001
<18.5	%	0.7	1.1		1.4	2.2	
18.5 – 24.9	%	25.4	35.4		23.9	33.9	
25.0 – 29.9	%	42.4	28.9		42.5	30.5	
≥ 30	%	31.5	34.6		32.2	33.4	
Race				0.008			0.001
Non-Hispanic White	%	72.9	74.1		80.8	74.6	
Non-Hispanic Black	%	8.6	10.7		9.7	9.6	
Hispanic and other	%	18.5	15.2		9.5	15.8	
Education				<.001			0.11
Less than 12th grade	%	13.4	9.5		24.7	23.7	
High School	%	25.3	22.5		26.1	31.4	
Some college	%	30.2	37.1		28.7	27.3	
College graduate or above	%	31.1	30.9		20.5	17.6	
Marital status				<.001			0.01
Live with someone	%	79.2	65.4		69.7	63.2	
Live alone	%	20.8	34.6		30.3	36.8	
Smoking				<.001			<.001
Never smoker	%	47.6	61.2		29.8	56.9	
Former smoker	%	27.8	21.2		48.5	25.4	
Current smoker	%	24.6	17.6		21.7	17.7	
Working hour duration (hours/week)	Mean (s.e.)	45.8 (0.5)	38.9 (0.4)	<.001	n.a.	n.a.	
Leisure time physical activity (LTPA)				0.01			0.18
Inactive	%	30.5	27.9		39.6	42.0	
Insufficiently Active	%	31.0	38.9		28.6	29.9	
Sufficiently Active	%	38.5	33.2		31.8	28.1	
Accelerometer measured moderate-to-vigorous physical activity (MVPA) (min/week)	Mean (s.e.)	57.1 (3.7)	45.6 (3.6)	0.004	41.1 (4.8)	28.8 (2.2)	0.02

a Weighted sample size to account for the complex survey design (including oversampling), survey non-response, and post-stratification in the NHANES study.

**Table 2:** Associations between Levels of Active Travel (transport) and Accelerometer Measured Moderate-to-Vigorous Intensity Physical Activity (min/week) from Unadjusted and Multivariable Linear Regression Models Among Employed and Unemployed Adults Aged 25 Years or Older from the NHANES (2003 - 2006).

	Accelerometer measured MVPA (min/week)			
	
	Men		Women	
Employed (Commuters, n=2,897)	Unadjusted Beta-coefficient (95% CI)	Adjusted^a^ Beta-coefficient (95% CI)	Unadjusted Beta-coefficient (95% CI)	Adjusted^a^ Beta-coefficient (95% CI)
Active Travel (Transport)				
Zero Active Travel	reference		reference	
Lower Level Active Travel (<90 min/week)	21.2 (3.0, 39.5)	8.15 (-8.5, 24.8)	18.1 (4.1, 32.2)	5.8 (-5.8, 17.4)
High Level Active Travel (≥90 minutes/week)	55.6 (29.9, 81.4)	40.8 (15.7, 65.9)	64.9 (36.2, 93.6)	57.9 (32.1, 83.7)
P for trend	<.001	0.002	<.001	<.001

Unemployed (n=2,283)	Unadjusted Beta-coefficient (95% CI)	Adjusted^b^ Beta-coefficient (95% CI)	Unadjusted Beta-coefficient (95% CI)	Adjusted^b^ Beta-coefficient (95% CI)
Active Travel (Transport)				
Zero Active Travel	reference		reference	
Lower Level Active Travel (<90 min/week)	19.5 (-5.3, 44.3)	10.0 (-14.8, 34.9)	8.9 (-8.9, 26.6)	2.1 (-12.7, 16.9)
High Level Active Travel (≥90 minutes/week)	61.3 (24.6, 97.9)	44.8 (9.2, 80.5)	18.9 (6.7, 31.1)	9.5 (-4.5, 23.6)
P for trend	0.001	0.01	0.001	0.18

a Adjusted for age, bmi, race and ethnicity, education, marital status, smoking status, work hour duration, and level of leisure-time physical activity,

b Adjusted for age, bmi, race and ethnicity, education, marital status, smoking status, and level of leisure-time physical activity,

## Summary Box

### What is already known on this subject?

Regular and sustained participation in physical activity is associated with better physical and mental health and is associated with healthy ageing in adults, but most adults in the US and other developed countries are not sufficiently active.
Efforts to increase population levels of leisure or occupational physical activity have yielded limited success particularly over the long term. Active transport (walking and bicycling for transportation) has the potential to transform routine daily living into an opportunity for physical activity.
Previous studies investigating the association between active travel and physical activity yield mixed results, probably due to lack of considering employment status and lack of sufficient sample in both gender groups. In addition, most studies used self-reported measures for physical activity assessment.

### What does this study add?

In a large representative sample of US adults, the distribution of active transport was similar between those who were employed and those who were not, such that 76.0% employed and 77.5% unemployed engaged in no active transport.
Employed men and women who used active transport achieved an additional 40.8 and 57.9 minutes of accelerometer measured MVPA a week, respectively. This amount of additional MVPA is associated with up to 20% reduction in all cause-mortality and mortality due to CVD and 13% reduction in cancer morality.
Among the unemployed adults, higher levels of active transport were associated with higher accelerometer measured MVPA in men (44.8 min/week MVPA, 95% CI: 9.2, 80.5) only. Additional strategies are likely required to promote physical activity among unemployed women.
